# Fifth Analysis of Cases of Tetanus Treated in Home Military Hospitals (100 Cases Observed in Dec. 1916 and Jan., Feb. and March, 1917)

**Published:** 1918-01

**Authors:** 


***Fifth Analysis of Cases of Tetanus Treated in Home Military Hospitals (100 Cases observed in Dec. 1916 and Jan., Feb. and March, 1917).*** By Sir David BRUCE. Abs. from the *Lancet*, Dec. 22, 1917.

B. notes that since his first report for 1914-15, the mortality from tetanus in the home hospitals has fallen from 57.7 o/o to 19 o/o. He notes also that the incubation period has tended to become longer, due to the more thorough use of preventive inoculation. Only 10 cases were reported with an incubation period of 10 days or less. The statistics afford no further evidence for or against the intrathecal method of injection, and none as to the size of doses. The statistics give no evidence of improvement in therapeutic treatment.

